# Genetic Predisposition and Inflammatory Bowel Disease

**DOI:** 10.4021/gr523w

**Published:** 2013-03-09

**Authors:** El-Tawil

**Affiliations:** a Department of Surgery, University Hospital Birmingham, Birmingham B15 2WB, United Kingdom

**Keywords:** Genetic predisposition, Familial aggregation, Environmental factors, Free species, Molecular genetics, Polymorphism, Inflammatory bowel diseases, Crohn’s disease, Ulcerative colitis

## Abstract

Published reports demonstrated finding of different susceptible mutant alleles in association with inflammatory bowel disease (CD/UC) in diseased individuals from different populations. It was then assumed that the existence of different associated mutant alleles in subjects with inflammatory bowel disease from different populations means different diseases. Whether this assumption is correct or false, this is the question that we are going to investigate.

## Introduction

Inflammatory bowel disease (IBD) represents a heterogeneous group of chronic disorders characterized by inflammation of gastrointestinal tract, typically with a relapsing and remitting clinical course of unknown etiology. Presumably, IBD develops with response to exogenous environmental factors only in persons with genetic predisposition.

Outraged production of free radicals and lack of afforded antioxidants are both quiet significant in subjects with inflammatory bowel diseases [1, 2] and it was described by me elsewhere [3, 4] how overproduction of reactive species could induce alterations in the genetic codes in a similar way as radiation can do. Likely as in the case of radiation, these free species would mostly affect the active genetic loci. But the activity of different genetic loci is determined by environmental factors as it has been explained above.

This study is aimed at investigating whether different associated mutant alleles in different affected subjects with IBD from different populations represent different diseases.

### Genetic mutation (alteration)

A mutation is defined as any change in the nucleotide sequence or arrangement of DNA. Mutation can be classified into three categories: mutations that affect the number of chromosomes in the cell (genome mutations), mutations that alter the structure of individual chromosomes (Chromosome mutations), and mutations that alter individual genes (Gene mutations) Genome mutations are alterations in the number of intact chromosomes (called aneuploidy) arising from errors in chromosome segregation during meiosis or mitosis. Chromosome mutations are changes involving only a part of a chromosome, such as duplications, deletions, insertions, and translocations, which can occur spontaneously or may result from abnormal segregation of translocated chromosomes during meiosis. Gene mutations are changes in DNA sequence, ranging from a change in as little as a single nucleotide to changes that may affect many thousands of base pairs, but always on a scale too small to be even seen with high-resolution cytogenetic analysis [5].

Mutant alleles are either null or amorph (an allel that produces no product); Hypomorph (an allele that produces a reduced amount or activity of product); Hypermorph (an allele that produces increased amount of activity of product); Neomorph (an allele with a novel activity or product) or Antimorph (an allele whose activity or product antagonizes the activity of the normal product) [6]. Loss of function mutations (amorph or hypomorph) most often produces recessive phenotypes. Sometimes, a nonfunctional mutant polypeptide interferes with the function of the normal allele in a heterozygous person, giving a dominant negative effect. But gain of function mutations usually causes dominant phenotypes, because the presence of a normal allele does not prevent the mutant allele from behaving abnormally. Often this involves a control or signaling system behaving improperly. Signaling when it should not, or failing to switch a process off when it should. Sometimes, the gain of function involves the product doing something novel. However, some other mutations cannot easily be classified as either loss or gain [6].

Inheritance of risk frequency can be determined by different factors including the position of the mutant allele and sex-threshold. For example, it was estimated that mutant alleles in the first or the second positions are more determinant than others [7]. Congenital pyloric stenosis is a representative example for the other determinant factor. Congenital pyloric stenosis is five times more common in boys than in girls. The threshold must be higher for girls than boys; therefore, relatives of affected girl have a higher average susceptibility than relatives of an affected boy [8].

The factors that govern the expression of pathogenic mutations are: the location of the mutation within the gene, the degree to which aspects of the aberrant phenotype are aberrant in the heterozygote, the degree to which expression of a mutant phenotype is influenced by other gene products, the proportion and nature of cells in which the mutant gene is present and the parental origin of the mutation [7].

### Mechanisms which affect the population frequency of alleles

Individuals within a population differ from each other. Much of the basis of such differences is due to inherited genetic variation. The frequency of any mutant allele in a population is dependent on a number of factors, Including natural selection (Natural selection is the process whereby some of the inherited genetic variation will result in differences between individuals regarding their ability to survive and reproduce successfully), random genetic drift (only a tiny fraction of the available gametes in any generation is ever passed to the next generation) and sequence exchanges between non-allelic sequences (individual genes in some gene families encode essentially the same product, but there may be sequence exchange occurring between the different gene copies) [7].

### Familial aggregation of a disease

Relatives share a greater proportion of their genes with one another than with unrelated individuals in the population. A primary characteristic of complex diseases’ inheritance is that affected individuals tend to cluster in families (Familial Aggregation). However, relatives may also share the same cultural attitudes and behaviors, diet, and environmental exposure. So, the reason(s) for familial aggregation may be genetic, environmental or both. When two related individuals in a family have the same disease, they are called “Concordant” for the disorder. But when only one member of the pair of relatives is affected and the other is not, the relatives are discordant for the disease. Diseases with complex inheritance result from the impact of environmental factors on individuals with certain genotypes. Discordance for phenotype between relatives who share a genotype at loci that predispose to disease can be explained if the unaffected individual has not experienced the other factors (environmental or chance) necessary to trigger the disease process and make it manifest Conversely, concordance for a phenotype may occur even when the two affected relatives have different predisposing genotypes, if the disease in one relative is a genocopy or phenocopy of the disease in the relative. Lack of penetrance and frequent genocopies and phenocopies all contribute to obscuring the inheritance pattern in multifactorial genetic disease [7].

## Evidences From Experimental Studies

Adamson RH and his colleagues [9] demonstrated that oral doses (0.3 g/kg) of Quinic acid were aromatized by gut flora to the extent of 20-70% in man, rhesus monkey, baboon and green monkey, but only 0-10% (usually nearly zero) in other species including spider monkey, squirrel monkey, capuchin, bush baby, slow Loris, tree shrew, dog, cat ferret, rabbit, rat, mouse, guinea pig, hamster, lemming, fruit bat, hedgehog and pigeon.

## Discussion

There is a common misunderstanding that led the majority of the physicians to believe that the finding of different mutant alleles in subjects with either Crohn’s disease and ulcerative colitis means different subgroups of the disease or different diseases. But the supporter of this ideology did not take into account the experience of the others. Based on experimental studies, it was concluded that a single genotype may give rise to many different phenotypes under varying circumstances of environment. This means that one environmental factor may act to exhibit various responses depending on the genotype of the organism, which determines the threshold of the responses [10]. Different populations have different life styles. This means that these different populations have different active alleles. The active alleles are the most affected loci by the effect of free species and they are thus more susceptible to the development of genetic mutations than others. This explains the reason for different mutant alleles in different subjects from different populations who have been affected with the same disease and presented more or less the same clinical features with few variations.

## References

[1] Rachmilewitz D (2008). Increased colonic nitric oxide level in active IBD. Scand J Gastroenterol.

[2] Rezaie A, Parker RD, Abdollahi M (2007). Oxidative stress and pathogenesis of inflammatory bowel disease: an epiphenomenon or the cause?. Dig Dis Sci.

[3] El-Tawil A (2010). Different prevalence of chronic-non-infectious diseases. Iran J Public Health.

[4] El-Tawil AM (2012). Pathogenesis of ulcerative colitis In: Ulcerative colitis: Symptoms, treatment and complications. Angelo Russo and Benedetta Gallo.

[5] Nussbaum RL, Mc Innes RR, Willard HF (2004). Genetic Variation in individuals: mutation and polymorphism. Genetics in medicine.

[6] Strachan T, Read AP (1999). Human Molecular Genetics 2.

[7] Strachan T, Read AP (1999). Human Molecular Genetics 2.

[8] Strachan T, Read AP (1999). Human Molecular Genetics 2.

[9] Adamson RH, Bridges JW, Evans ME, Williams RT (1970). Species differences in the aromatization of quinic acid in vivo and the role of gut bacteria. Biochem J.

[10] Burdon RH (1999). Gene and the environment.

